# Laser Microinterferometry for API Solubility and Phase Equilibria: Darunavir as a Case Example

**DOI:** 10.3390/pharmaceutics17070875

**Published:** 2025-07-03

**Authors:** Veronika Makarova, Mark Mandrik, Sergey Antonov

**Affiliations:** 1 A.V. Topchiev Institute of Petrochemical Synthesis, Russian Academy of Sciences, Leninsky pr. 29, 119991 Moscow, Russia; 2 Department of Pharmaceutical Technology, Federal State Autonomous Educational Institution of Higher Education, I.M. Sechenov First Moscow State Medical University (Sechenov University) of the Ministry of Health of the Russian Federation, 8-2 Trubetskaya str., 119991 Moscow, Russia

**Keywords:** thermodynamic solubility, phase diagrams, laser microinterferometry, dissolution kinetics, active pharmaceutical ingredient, early-stage drug development, amorphous darunavir, Hansen solubility parameters

## Abstract

**Background:** The solubility and phase behavior of APIs are crucial for the development of medicines and ensuring their stability. However, conventional experimental approaches often do not allow for the precise determination of phase transitions and solubility limits, especially for poorly soluble compounds. **Purpose:** The aim of this study was to demonstrate the possibility of using the laser microinterferometry method, traditionally used to define the phase equilibria of polymer systems, to determine the thermodynamic solubility of the APIs. **Methods:** Using laser microinterferometry, the thermodynamic solubility and phase behavior of amorphous darunavir were determined in various pharmaceutical solvents, including vaseline and olive oils, water, glycerol, alcohols (methanol, ethanol, isopropanol), glycols (propylene glycol, polyethylene glycol 400, polypropylene glycol 425, polyethylene glycol 4000), and ethoxylated polyethylene glycol ether obtained from castor oil in the temperature range of 25–130 °C. Dissolution kinetics was estimated at 25 °C. Hansen solubility parameter calculations were also performed for comparison. **Results:** Darunavir is practically insoluble in olive and vaseline oils. In water and glycerol, an amorphous equilibrium with an upper critical solution temperature was observed, and phase diagrams were constructed for the first time. In alcohols, glycols, and ethoxylated polyethylene glycol ether obtained from castor oil, darunavir showed high solubility, accompanied by the formation of crystalline solvates. Kinetic evaluation showed that the dissolution rate of darunavir in methanol is four times faster than in ethanol and thirty times faster than in isopropanol. Comparison of the obtained data with previously published and calculated values of solubility parameters demonstrates a good correlation. **Conclusions:** Laser microinterferometry has been demonstrated as a potential tool for determining the thermodynamic solubility of APIs. This method allows for directly observing the dissolution process, determining the solubility limits, and detecting phase transitions. These studies are necessary for selecting appropriate excipients, preventing the formation of undesirable solvates and predicting formulation stability, which are all critical factors in early-stage drug development and pharmaceutical formulation design.

## 1. Introduction

The solubility of an active pharmaceutical ingredient (API) in water and various excipients is a key physicochemical parameter that characterizes the API and determines the entire process of drug development, including the choice of the dosage form and the production technology. In the case of APIs belonging to classes II and IV of the biopharmaceutical classification system (BCS), improving solubility is one of the main ways to increase their bioavailability. Therefore, understanding the concept of the dissolution process and choosing a solubility measurement method is important.

Traditionally in chemistry, solubility is defined as the maximum amount of a substance that can be dissolved in a given volume of solvent at certain temperatures and pressures to form a molecular dispersion representing a thermodynamic equilibrium. However, in pharmaceutical literature, two types of solubility are fundamentally distinguished, depending on the measurement method: kinetic solubility and thermodynamic solubility [1]. This dual terminology reflects practical needs—rapid screening versus the complexity of measuring equilibrium in unstable or polymorphic APIs.

Kinetic solubility is commonly assessed by the direct agitation of API powder with solvent followed by supernatant quantification, intrinsic dissolution rotating disk tests, or indirect precipitation onset methods—such as turbidimetry, nephelometry, or potentiometric titration—after preliminary dissolution in an organic vehicle. Although widely used for rapid screening, these approaches frequently overestimate solubility and exhibit poor inter-laboratory reproducibility owing to methodological variability [2,3,4,5,6,7,8,9,10,11,12,13,14,15].

Thermodynamic solubility is typically measured with the saturation shake-flask (SSF) procedure, wherein excess API is equilibrated with solvent under controlled agitation and the saturated phase is analyzed after separation. Miniaturized variants and automated circulation systems have improved throughput [6,16,17,18,19,20,21,22,23,24,25,26,27]; nevertheless, SSF remains labour-intensive, time-consuming, and generally restricted to single-temperature determinations.

Alternative techniques for determining equilibrium solubility include microdissolution (µDISS), which enables the in situ UV spectroscopic monitoring of concentration profiles using immersion fiber-optic probes [28], and the facilitated dissolution method (FDM), which employs water-immiscible solvents to enhance dissolution rates [29,30]. For ionizable APIs, pH metric titration offers a thermodynamically relevant estimate by exploiting titration curve shifts due to undissolved solids [3,31]; the dissolution titration template (DTT) further accelerates this process via a three-component model [32]. More recent innovations include quartz crystal microbalance (QCM) analysis, which assesses solubility and nucleation upon thermal cycling [33], and single-particle approaches such as optical image-based analysis (SPA), which trap particles hydrodynamically for the optical monitoring of dissolution equilibrium at the microscale [34,35]. Despite these advancements, most methods remain limited by their time requirements, single-temperature data points (typically 37 °C), and inability to provide dissolution kinetics.

These limitations highlight a clear demand for more effective and scientifically robust methods for determining thermodynamic solubility. In the context of early-stage pharmaceutical development, it is particularly important to employ approaches suitable for screening that enable accurate equilibrium solubility assessment over a wide temperature range. Such methodologies should ensure the minimal consumption of APIs, be applicable to various systems, and provide reproducible and interpretable results. Moreover, the ability to assess temperature-dependent solubility profiles, as well as obtain complementary data on diffusion processes, would significantly contribute to rational formulation design and optimization. Consequently, the advancement of novel analytical techniques meeting these criteria represents a critical objective in contemporary pharmaceutical science.

The process of dissolution, as is known, is directly related to diffusion, i.e., the spontaneous distribution of molecules driven by concentration gradients. In this study, laser microinterferometry (wedge microinterferometry), a diffusion-based technique, is introduced into pharmaceutical research as a novel approach for determining the thermodynamic solubility of active pharmaceutical ingredients (APIs). Previously established in polymer science and crystallization studies [36,37,38,39,40,41,42,43,44,45,46,47,48,49,50], this method relies on the formation of interference patterns in a thin wedge-shaped gap, allowing for the precise visualization and quantification of concentration gradients in the diffusion zone.

From these optical profiles, equilibrium solubility can be directly determined at various temperatures, supporting the construction of solubility curves and phase diagrams. Thus, the laser microinterferometry method is characterized by minimal sample consumption, rapidity and high information content. It can be used to study the thermodynamic solubility of APIs both in water and buffer solutions, organic solvents, excipients of oligomeric and polymeric nature, and in a wide temperature range, which is of great interest from the point of view of pharmaceutical technology, especially for early-stage pharmaceutical development

Darunavir, an antiretroviral drug for the treatment of HIV and the prevention of AIDS, was used as an API in this study. It is a non-peptide protease inhibitor effective against wild-type HIV, and an important component of highly active antiretroviral therapy [51]. Darunavir has low water solubility and belongs to BCS class II, having a bioavailability of about 37%, making it a relevant candidate for solubility studies [52,53]. The thermodynamic solubility of darunavir was studied using laser microinterometry in a wide range of solvents most commonly used in the pharmaceutical industry in order to demonstrate the possibility of using this method in pharmaceuticals and to directly study the phase equilibrium of various darunavir–solvent systems. The obtained experimental data were compared with those known from the literature. In addition, solubility parameters of darunavir and solvents were calculated using HSPiP version 5.4.08 software to confirm the accuracy of measurements.

### Fundamentals of Laser Microinterferometry

**Figure 1:**
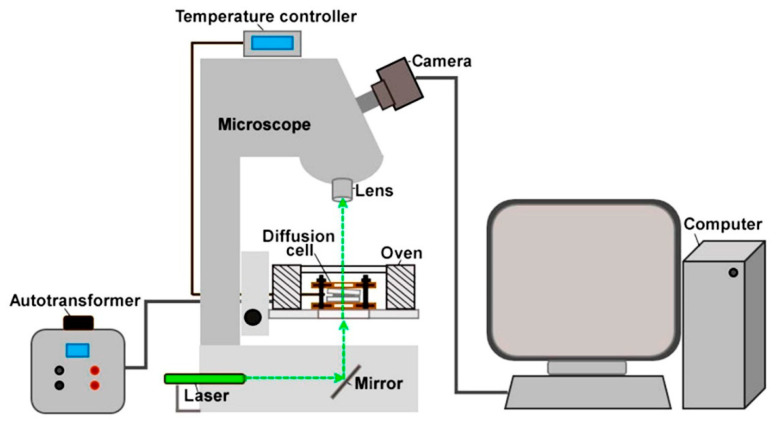
Scheme of laboratory interferometer setup.

The solubility of the API was determined using laser microinterferometry, which has been widely used to study the phase equilibrium of binary polymer systems [36,37,38,39,40,41,42,43,44,45,46]. The laboratory interferometer setup (Figure 1) included a microscope, to the object table of which an electric mini-oven was attached, into which a diffusion cell with the components being studied was placed. Smooth heating/cooling of the oven was carried out using a laboratory autotransformer, and the temperature in it was controlled using a temperature controller. The oven had a transparent bottom and a top cover made of heat-resistant glass. The diffusion cell consisted of two glass plates coated on one side with a thin layer of metal. The components under study were placed side by side between the plates, and the structure was fixed using metal clamps. The diffusion cell was illuminated using a laser, and the image was received using a video camera connected to a computer through a lens.

**Figure 2:**
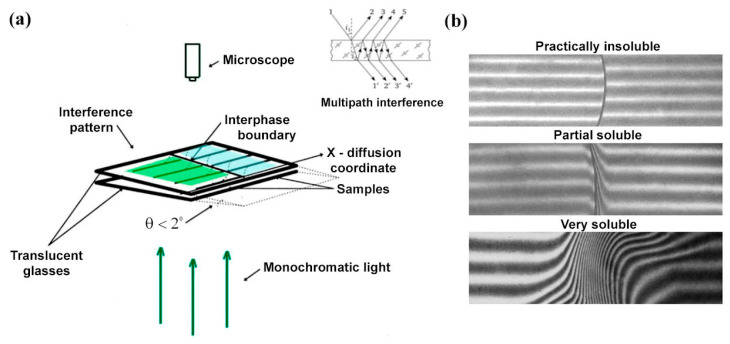
Experimental scheme in the laser microinterferometry method (**a**) and types of interferograms for systems with different solubilities (**b**).

Measurements in laser microinterferometry are based on multipath interference from two surfaces of plane-parallel plates, forming a small angle θ < 2° (wedge) between them and allowing for the in situ observation of interdiffusion process of components in a binary system (Figure 2a). Film samples (powders and liquids can also be used) are placed side by side between two glass plates, the inner surfaces of which are coated with a translucent layer of metal (Ag, Ni-Cr alloy, etc.) to enhance reflectivity. The distance between the plates varies between 60 and 120 μm. When a beam of monochromatic light (laser) passes through the gap of variable thickness, an interference pattern is formed. It consists of two systems with interleaved light and dark bands separated by an interphase boundary. As a result of mutual penetration of components near the interphase boundary, concentration gradients appear. This leads to a change in optical density and, as a consequence, to bending of interference bands. The evolution of the band shape provides information about the intensity of components penetration and the distribution of their concentrations in the diffusion zone.

The main types of interferograms observed during the experiment are presented in Figure 2b. The absence of any penetration of components is reflected in the constancy of the interference bands: they remain perpendicular to the interphase boundary. From the point of view of pharmaceutical terminology, this type of interferogram can be attributed to the case when the API is practically insoluble in a given solvent. With a limited penetration of components, interference bands near the interphase boundary are bent. Here, we can consider partially soluble components and the presence of amorphous equilibrium in a system with an upper/lower critical mixing temperature (UCTS/LCST). Depending on the value of the maximum dissolution concentration, the situation can be described in pharmaceutical literature using terms such as very slightly soluble, slightly soluble, sparingly soluble, soluble, and freely soluble. Unlimited dissolution of components leads to the disappearance of the interphase boundary and the continuity of interference bands. Such interferograms are typical for the case when the API is very soluble in a given solvent.

The processing of interferograms and the construction of concentration profiles in the interdiffusion zone were carried out based on refractometry [36,37,38]. According to the laws of geometric optics, each interference band is a geometric place of dots in which the optical density of the wedge is the same:

(1) $$\nu \lambda =2{\left(nl\right)}_{i}$$

where *ν*—proportionality coefficient, *λ*—wavelength of light, *n*—refractive index of the medium, and *l*—local thickness of the wedge at the place of the *i*-th band. At *n* = const (one component), a system of bands parallel to the edge of the wedge is formed. In the case of a binary system with different refractive indices of components (*n*_1_ and *n*_2_), two systems of interference bands are formed, separated by an interphase boundary. During the diffusion of one component into the medium of another, a refractive index gradient appears in it, which leads to an increase in the density of interference bands and a change in their position relative to the edge of the wedge (bends). In this case, each interference band continues to describe a contour with a constant optical density, keeping the multiplication ${\left(nl\right)}_{i}$ constant.

To determine the concentration profile in the interdiffusion zone, a line of equal thickness is drawn on the interferograms $\underline{l}=const$, along which the change in optical density depends only on the change in the refractive index:

(2) $$\nu \lambda =2∆n\underline{l}$$

In the case of interferograms where all interference bands are resolved (Figure 2b, very soluble), the change in refractive index per band can be calculated using the following equation:

(3) $$∆n=\left(n_{1}-n_{2}\right)/N$$

where *N* is the number of bands on a line of equal thickness between points with known *n*_1_ and *n*_2_ (initial components). The values of Δ*n* from Equations (2) and (3) are in good agreement with each other, so the number of bands *N* can be expressed as follows:

(4) $$N=\frac{\left(n_{1}-n_{2}\right)}{∆n}=\frac{2\underline{l}\left(n_{1}-n_{2}\right)}{\lambda }$$

Taking $\underline{l}$, equal to the thickness of the wedge gap in the first approximation (due to its change at *θ* < 2° no higher than 2–5%), it is possible to determine the number of bands in the case when the interference bands are partially resolved and an interphase boundary is present (the case of partial soluble). To move to the distribution curves of concentration by distance, it is proceeded from a linearity of the concentration dependence of the refractive index, and the change in concentration per band ∆*φ* and the volume fraction of the component *φ* were taken to be equal:

(5) $$∆\varphi =1/N\;\;and\;\;\varphi =∆\varphi N_{i}$$

**Figure 3:**
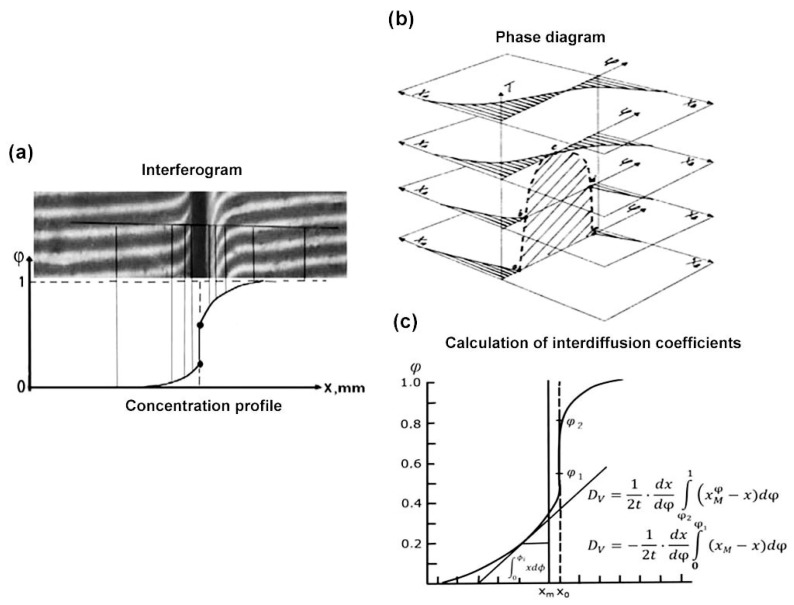
Schemes for interferograms processing (**a**), a phase diagrams constructing (**b**), and interdiffusion coefficients calculating (**c**).

Therefore, knowing the values of the refractive indices of the original components and determining the position of the crossing points of the interference bands with a line of equal thickness, we can obtain the concentration profile in the interdiffusion zone (Figure 3a). In the case of a “partial soluble”, the equilibrium concentration values near the interphase boundary were obtained by constructing concentration profiles on both sides of the components.

Conducting experiments in the step heating/cooling mode and determining concentration profiles at different temperatures allows us to construct phase diagrams (Figure 3b). Experiments conducted at constant temperatures in time make to obtain information about the kinetics of the diffusion process. In particular, from concentration profiles, the interdiffusion coefficients can be calculated based on Fick’s second low using the Matano–Boltzmann method [36,37,38,39,41,43,44,45,54,55]. The basic equations are shown in Figure 3c. There are also other ways.

Thus, the laser microinterferometry method allows for determining the thermodynamic solubility of substances, constructing the phase diagrams of binary systems, and investigating the kinetics of the diffusion process. In addition, it is characterized by compact equipment, low amount of the studied substances, and short measurement time. The limitations of the method are related to the need for optical transparency of at least one of the studied components and a sufficient difference in their refractive indices (not less than 10^−3^).

## 2. Materials and Methods

### 2.1. Materials

Amorphous darunavir purchased from Henan Tianfu Chemical Co., Ltd. (Zhengzhou, China) with a water content of 0.4% was selected as the API.

A wide range of solvents contained distilled water; glycerol (USP/EP grade, Scharlab, Sentmenat, Spain); alcohols: methanol (HPLC grade, Chimmed, Moscow, Russia), ethanol (pure, Merck, Darmstadt, Germany), and isopropanol (pure, Chimmed, Moscow, Russia); glycols: 1,2-propylene glycol (PG) (USP/EP grade, BASF, Ludwigshafen, Germany), polyethylene glycol 400 (PEG 400) (Pharma grade, Merck, Darmstadt, Germany), polypropylene glycol 425 (PPG 425) (Acros Organics, Geel, Belgium), and polyethylene glycol 4000 (PEG 4000) (USP/EP grade, Clariant AG, Muttenz, Switzerland); oils: vaseline oil (USP/EP, PanReac, Castellar del Vallès, Spain) and olive oil (refined, Himpitertorg Group, St. Petersburg, Russia); as well as ethoxlyated polyethylene glycol ester made from castor oil (PEG-40 HCO) (BASF, Ludwigshafen, Germany).

### 2.2. Experimental Methods

#### 2.2.1. X-Ray Diffraction

The crystallinity of darunavir was investigated by X-ray diffraction on a Rigaku Rotaflex D/max-RC instrument (Rigaku, Akishima, Japan) using CuKα radiation (*λ* = 0.154 nm). X-ray diffraction patterns of the samples were recorded in the 2*θ* angular range of 3–55° with a step of 0.02° at a recording rate of 2°/min.

#### 2.2.2. Differential Scanning Calorimetry and Thermogravimetric Analysis

The phase transition temperatures of darunavir were studied by thermogravimetric analysis (TGA) and differential scanning calorimetry (DSC) on Thermal Analysis System TGA/DSC 3+ (Mettler Toledo, Greifensee, Switzerland). Analyses were carried out in a nitrogen environment with a flow rate of 50 mL/min in an aluminum oxide crucible with a volume of 150 μL in the temperature range 25–1000 °C with a constant heating rate of 10 K/min.

#### 2.2.3. Laser Microinterferometry

The solubility of darunavir was determined using a laboratory interferometer consisting of the microscope MBI 15U4.2 (LOMO, St. Petersburg, Russia) with a 3.5× lens, an electric mini-oven (TIPS RAS, Moscow, Russia), a laboratory autotransformer 2000VA SUNTEK SK2.1 LTR2000 (SUNTEK, Shenzhen, China), a temperature controller TRM 500 (OVEN, Moscow, Russia) with a measurement error of ±0.5%, a modular laser KLM-A532-15-5 (FTI-Optronic, St. Petersburg, Russia) with a wavelength of 532 nm, and a digital video camera Levenhuk C800 NG (Levenhuk, Tampa, FL, USA).

Film samples of darunavir with a thickness of 60–80 μm were prepared by briefly pressing darunavir powder at 80 °C. The diffusion cell was collected as follows. The darunavir sample and spacers regulating the gap thickness were placed between 20 mm × 25 mm × 4 mm glass plates with Ag coating, and the structure was fixed using metal clamps so that the interference bands were oriented parallel to the edge of the wedge and perpendicular to the diffusion front. Then, the diffusion cell was thermostated to a set-point temperature and a solvent was added to the gap. The moment of contact of the components was taken as the beginning of the diffusion process. Heating and cooling of diffusion cells were carried out stepwise with an exposure of at least 20 min at each step. The temperature range varied within 25–130 °C depending on the thermal properties of initial components. The processing of the interferograms did not differ from traditional ones and is described in Section 2.

#### 2.2.4. Refractometry

**Figure 4:**
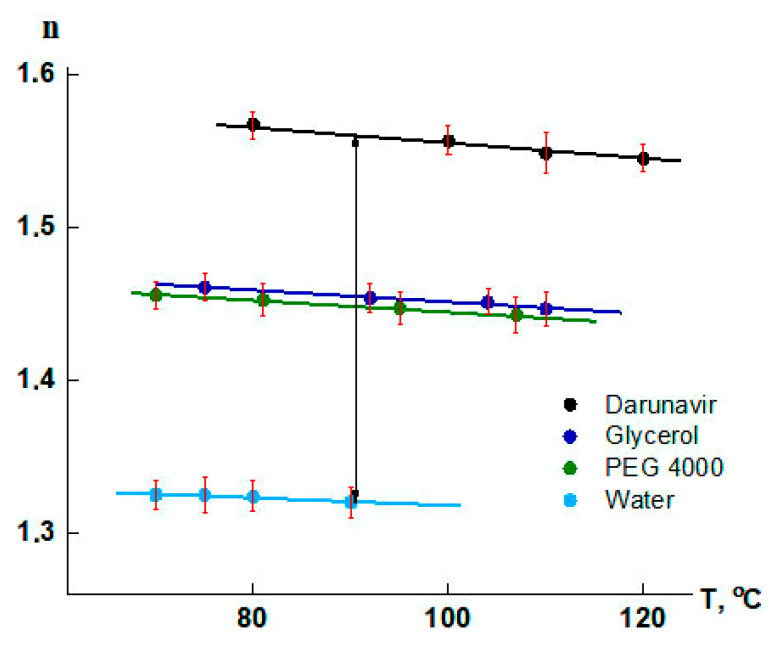
Temperature dependence of the refraction indices of darunavir, water, glycerol, and PEG 4000.

The refractive indices of the initial components were determined using IRF-22 refractometer equipped with an oil thermostat at 25 °C. The results are presented in Table 1. For the processing of the interferograms of darunavir–water, darunavir–glycerol, and darunavir–PEG 4000 systems, additional measurements of the refraction indices were carried out in a temperature range 80–120 °C (Figure 4). The refractive index value of darunavir at 25 °C was determined by extrapolation of the temperature dependence.

#### 2.2.5. HSPiP Software-Based Solubility Prediction

Using Dr. Yamamoto Hiroshi’s Y-MB neural network module and HSPiP (Hansen Solubility Parameters in Practice) version 5.4.08, the Hansen solubility parameters for darunavir and solvents were calculated or taken from the dataset [56,57]. The simplified molecular string input syntax code was used as the input to the built-in group contribution method in HSPiP.

## 3. Results and Discussion

### 3.1. Characteristics of the Structure and Thermal Transitions of Darunavir

**Figure 5:**
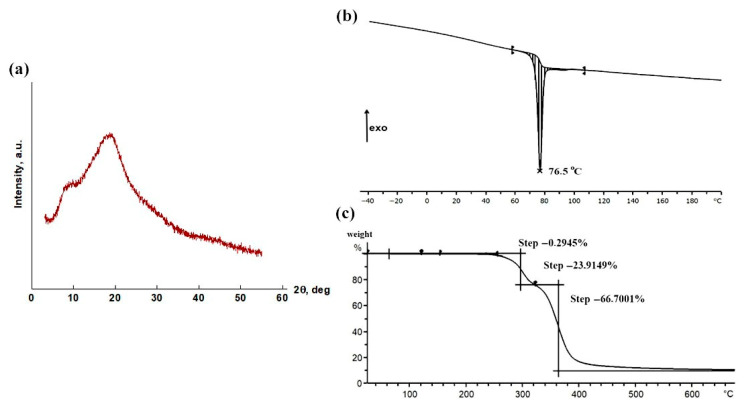
X-ray diffraction pattern (**a**), DSC heating thermogram (**b**), and temperature dependence of weight loss (**c**) of darunavir.

The structure and phase transition temperatures of amorphous darunavir were studied using X-ray diffraction analysis, differential scanning calorimetry, and thermogravimetric analysis. According to the obtained diffraction pattern, the structure of darunavir is amorphous (Figure 5a). However, two maxima are distinguished with angular positions of 8.5 and 19°. Their existence indicates the presence in the amorphous darunavir of short-range-order regions with a local periodicity of 10.5 and 4.7 Å (according to the Wulff–Bragg formula). Taking these regions as possible crystalline and deconvolving the diffraction pattern using 4 pseudo-Voigt functions, we estimated the degree of crystallinity to be 10.5% (the proportion of the sum of the peak areas relative to the total area of all peaks.).

In accordance with DSC data, an endothermic effect of −10.4 J/g appeared in the temperature range of 70–80 °C (Figure 5b). It is probably associated with the melting of the above-mentioned crystalline phase. Using the HSPiP version 5.4.08 program [56,57], the theoretical value of the enthalpy of melting of fully crystalline darunavir was determined to be 155.2 J/g. In accordance with this value and the DSC data, the crystallinity of amorphous darunavir was 6.7%, which correlates well with the X-ray structural analysis data.

Thermogravimetric data show that the darunavir weight loss, i.e., the beginning of the process of its active decomposition, occurred in three stages, starting from temperatures above 200 °C (Figure 5c). This fact makes it possible to obtain film samples of darunavir for laser microinterferometry experiments by the short-term pressing of darunavir powder at 80 °C, and to carry out investigations in the temperature range of 25–130 °C. The decomposition of darunavir will not occur.

### 3.2. Dissolution of Darunavir in Solvents

#### 3.2.1. Practically Insoluble

**Figure 6:**
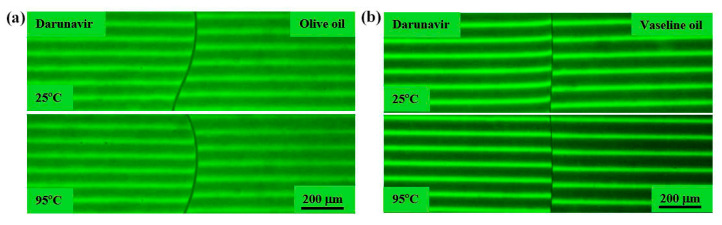
Interferograms of the systems darunavir–olive oil (**a**) and darunavir–vaseline oil (**b**) at 25 and 95 °C. The interference bands remained unchanged in all ranges of the study temperatures. Darunavir was practically insoluble in the oils.

The interaction of darunavir with oils was studied in the temperature range of 25–130 °C. No changes in the diffusion zone were observed when olive oil came into contact with the darunavir film sample (Figure 6a). The interference bands on both sides of the interphase boundary remained unchanged. An increase in temperature did not change their position, indicating no penetration of the components into each other. A similar picture was observed in the case of the darunavir–vaseline oil system (Figure 6b). Thus, it can be concluded that darunavir is practically insoluble in the oils studied.

Therefore, the absence of any observable diffusion or interaction in the studied temperature range indicates that these oils are not suitable as solvents for darunavir in pharmaceutical formulations.

#### 3.2.2. Partial Soluble

##### Darunavir–Water System

**Figure 7:**
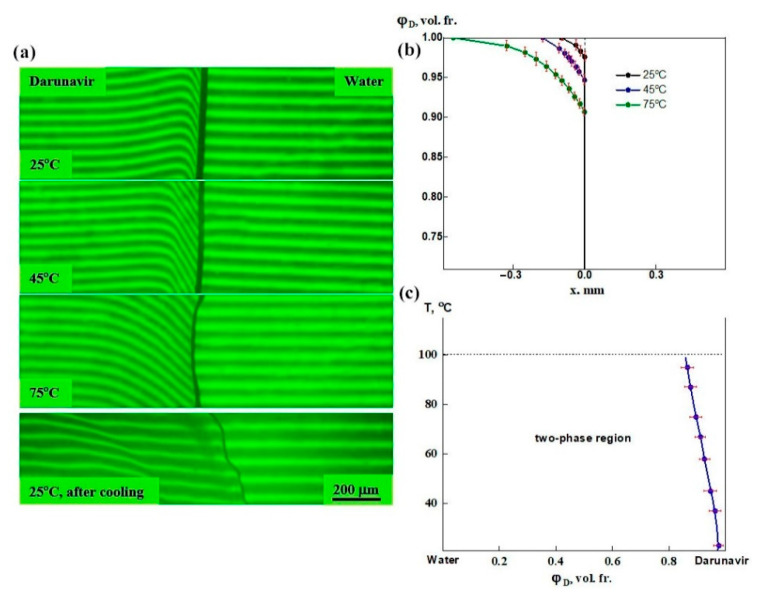
Interferograms of the darunavir–water system (**a**), concentration profiles of darunavir in the diffusion zone at different temperatures (**b**), and phase diagram of the darunavir–water system (**c**). The interference bands on the darunavir side bend, intensifying at increasing temperature, and remain unchanged on the water side. Darunavir is practically insoluble in water, but swells in it. After cooling, phase separation occurred. The phase equilibrium of the system refers to systems with UCST.

For the darunavir–water system, the experiments were carried out in the temperature range of 25–95 °C to avoid boiling of the water. After contact of the darunavir film sample with water at room temperature, a slight bend in the interference bands appears along the interphase boundary on the darunavir side, indicating the penetration of water into darunavir (Figure 7a). At the same time, the interference bands remained unchanged on the water side. This indicates that darunavir is practically insoluble in water, but swells slightly: it is hygroscopic. An increase in temperature leads to an increase in the bending of the interference bands, i.e., an increase in water absorption. Further cooling of the diffusion cell to room temperature leads to a decrease in the bending of the bands and the appearance of a dark area, whereby excess absorbed water is released. Repeated heating leads to its disappearance, indicating the reversibility of the process. Such a character of the change in the interference pattern indicates the presence of an amorphous equilibrium in the system with an upper critical solution temperature (UCST).

According to refractometry data (Figure 4), the difference in refraction indices between darunavir and water is 0.24, which corresponds to the formation of 54 bands in the interdiffusion zone. Based on refractometric data, concentration profiles were constructed at different temperatures (Figure 7b). The values of the equilibrium concentrations of the system components formed a phase diagram, i.e., a binodal (Figure 7c). The left branch of the binodal merges with the ordinate axis. Darunavir is practically insoluble in water, and only slightly swells in it. According to the phase diagram, darunavir was able to absorb 2.7–14 vol.% water in the temperature range of 25–95 °C. It is known that the amorphous form of darunavir is hygroscopic and the weight gain is 2.617% at 25 °C [58]. This is in good agreement with the results of the present study.

These findings confirm that water, even at elevated temperatures, is unsuitable as a solvent for darunavir in pharmaceutical applications. This highlights the need for solubility enhancement strategies to ensure sufficient bioavailability in drug formulations.

##### Darunavir–Glycerol System

**Figure 8:**
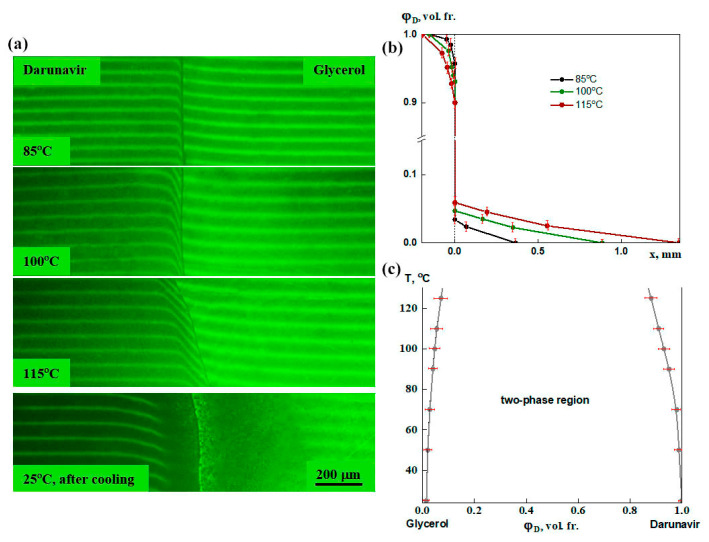
Interferograms of the darunavir–glycerol system (**a**), concentration profiles of darunavir in the diffusion zone at different temperatures (**b**), and phase diagram of the darunavir–glycerol system (**c**). On both sides of the interphase boundary, there are bends in the interference bands, which intensify with increasing temperature. Darunavir is partially soluble in glycerol. After cooling, phase separation occurred. The phase equilibrium of the system refers to systems with UCST.

The solubility of darunavir in glycerol was studied in the temperature range of 25–130 °C. Noticeable changes in the interference pattern began to occur at temperatures above 80 °C. Weak bends in the interference bands were observed on both sides of the interphase boundary, which increased with increasing temperature (Figure 8a). After cooling, dark areas were formed in the region of interdiffusion, i.e., phase separation occurred. This indicates the presence of an amorphous equilibrium with the UCST in the system.

According to refractometry data (Figure 4), the difference in refractive indices between darunavir and glycerol was 0.11, which corresponds to the formation of 21 bands in the interdiffusion zone. Based on these data, concentration profiles were constructed at different temperatures (Figure 8b). The found equilibrium concentrations of the components formed a phase diagram of the system, i.e., a binodal with UCTS (Figure 8c), the value of which lies in the region of high temperatures. In the temperature range of 25–80 °C, the binodal branches were close to the ordinate axis: the solubility of the components is low. Solubility values increased above 80 °C. This was likely due to the improved mobility of darunavir molecules. In general, it can be concluded that in the temperature range of 25–130 °C, darunavir could absorb 0.4–1.2 vol.% of glycerol, while 1.4–7.0 vol.% of darunavir dissolved in glycerol.

Based on these findings, glycerol demonstrates limited capacity to dissolve darunavir at typical formulation temperatures.

#### 3.2.3. Very Soluble

##### Darunavir–Alcohol Systems

**Figure 9:**
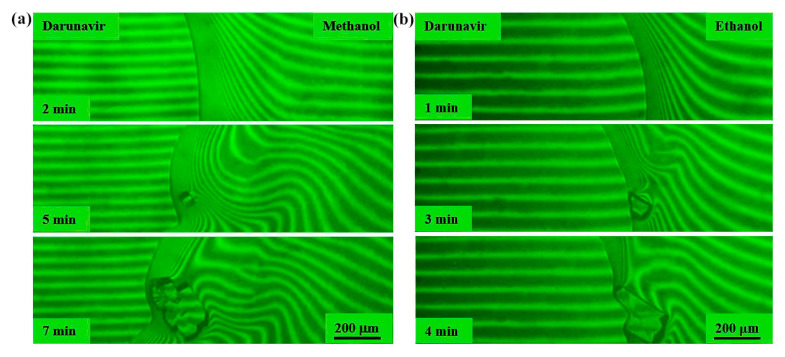
Interferograms of the darunavir–methanol (**a**) and darunavir–ethanol (**b**) systems at 25 °C. There is no interphase boundary. Darunavir is very soluble in methanol and ethanol. Crystal solvates were formed over time.

**Figure 10:**
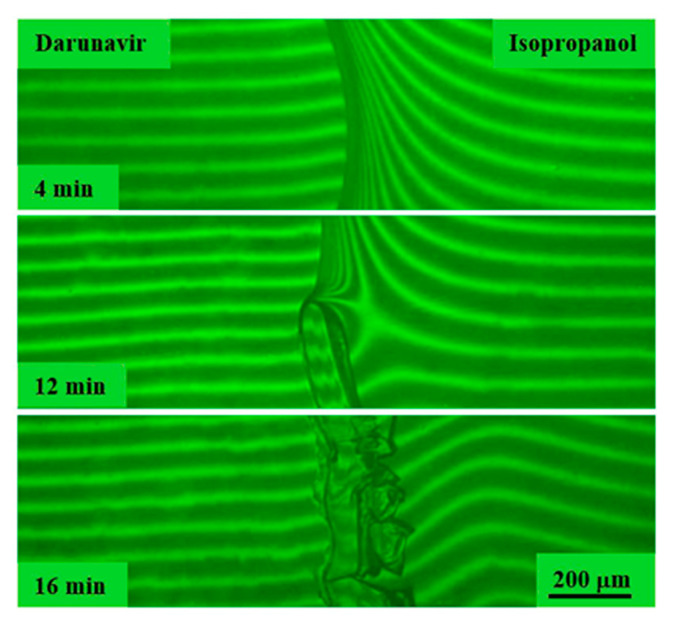
Interferograms of darunavir-isopropanol systems at 25 °C. There is no interphase boundary. Darunavir is very soluble in isopropanol. Crystal solvates were formed over time.

Dissolution studies of darunavir in alcohols were carried out in two modes: holding for an hour at 25 °C and heating within the intense boiling points of the solvent at 25–55 °C (in the case of methanol) and 25–70 °C (in the case of ethanol and isopropanol). After the film sample of darunavir came into contact with methanol, ethanol, or isopropanol at 25 °C, many interference bands formed on the alcohol side (Figure 9 and Figure 10). The visible optical boundary was not an interphase boundary, it moved over time: the dimensions of the film sample of darunavir decreased, i.e., darunavir is very soluble in alcohols. An increase in temperature accelerates the movement of the boundary, i.e., it promotes a faster dissolution process. The optical effect of boundary displacement in the direction of diffusion penetration during diffusion of solvents into a polymer is known [36]. A detailed study of such a boundary showed that it is localized at the solvent front in the concentration range of 2–4 vol.%, and its occurrence is associated with a sharp change in the refractive index gradient within a very narrow zone.

After some time (minutes) from the beginning of the dissolution process, crystals began to form and grow in the diffusion region in all systems. At the same time, dissolution slows down—the bends of the interference bands are leveled. An increase in temperature helped accelerate the dissolution process, melt the crystals, and evaporate the solvent.

It has been previously shown that amorphous darunavir can easily form crystal solvates, i.e., products of its interaction with various solvents [59,60]. In our case, we were probably observing the formation of methanolates, ethanolates and isopropanolates, respectively. The melting points of these crystal solvates were different. When the diffusion cell was heated, methanolates began to melt above 67 °C, ethanolates above 108 °C, and isopropanolates above 114 °C. This correlates with data from [60], in which, according to DSC data, methanolates have a melting peak at 65 °C, ethanolates at 110 °C, and isopropanolates at 115 °C. Thus, laser microinterferometry makes it possible to visually observe not only the dissolution process, but also the formation of crystals and their melting. This is interesting from the point of view of assessing the stability of the drug being developed, especially in the case of accelerated stability testing, when it is necessary to demonstrate that the amorphous substance used to obtain the drug does not crystallize spontaneously.

Thus, methanol and isopropanol are not suitable for pharmaceutical applications due to the formation of crystal solvates, which can lead to the presence of toxic alcohols upon administration. Ethanol, on the other hand, may be acceptable, especially given the known use of darunavir ethanolate as an API. However, in formulations containing residual ethanol, special attention should be paid to the possibility of recrystallization of amorphous darunavir into the ethanolate form, which may impact the stability and therapeutic performance of the final drug product.

##### Darunavir–Glycol Systems

**Figure 11:**
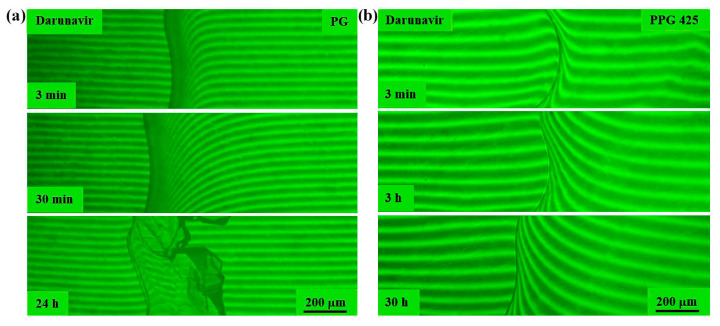
Interferograms of the systems darunavir–PG (**a**) and darunavir–PPG 425 (**b**) at 25 °C. There is no interphase boundary. Darunavir is very soluble in PG and PPG. Crystal solvates were formed in the darunavir–PG system over time.

**Figure 12:**
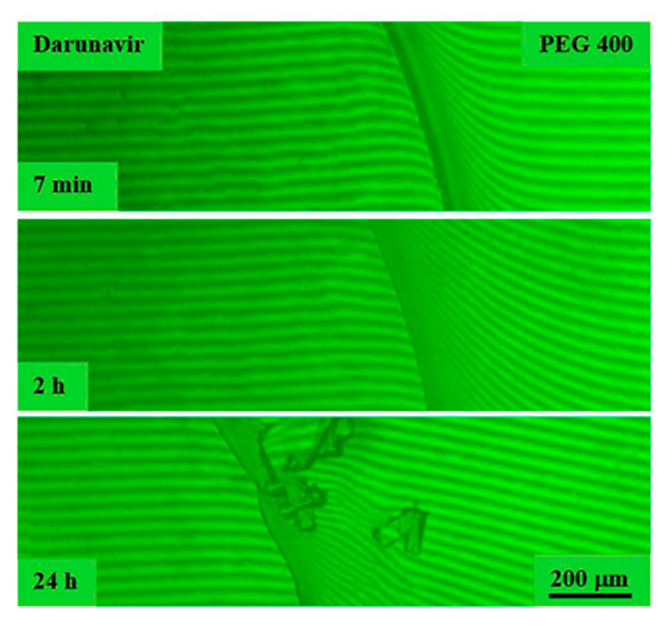
Interferograms of the darunavir–PEG 400 system at 25 °C. There is no interphase boundary. Darunavir is very soluble in PEG 400. Crystal solvates were formed over time.

Dissolution studies of darunavir in glycols were carried out in two modes: holding for 30 h at 25 °C and heating within the temperature range of 25–130 °C. After the film sample of darunavir came into contact with PG and PEG 400, many interference bands were formed in the interdiffusion zone on the solvent side (Figure 11a and Figure 12), similar to darunavir–alcohol systems. In the case of the darunavir–PPG 425 system, the number of interference bands was smaller, but the size of the darunavir film sample also decreased with time (Figure 11b). Darunavir is very soluble in glycols. The dissolution process continued longer compared to alcohols, and took hours. An increase in temperature accelerated the process.

After more than 20 h in the interdiffusion zone, just as in the case of alcohols, the nucleation and growth of crystal solvates occurred. An increase in the molecular weight of the solvent apparently slows down the process significantly. The formation of crystals for the darunavir–PPG 425 system during the experiment (30 h) was not observed.

**Figure 13:**
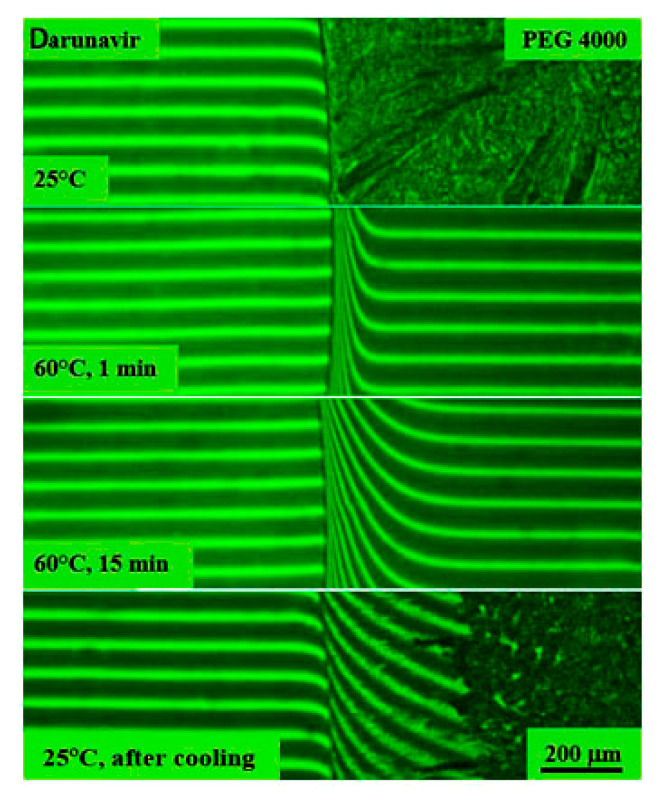
Interferograms of the darunavir–PEG 4000 system. There is no interphase boundary above 60 °C. Darunavir is very soluble in PEG 4000. Below 60 °C, the darunavir–PEG 4000 system is characterized by crystalline equilibrium.

In the case of the darunavir–polyethyleneglycol 4000 system, experiments were carried out starting from 60 °C, since at room temperature, PEG 4000 is in a crystalline state, and its melting point is about 58 °C. Holding for an hour was also carried out at 60 °C (Figure 13). When the components came into contact, many interference bands were formed on the solvent side, similar to the systems described above, and the sizes of the film sample of darunavir gradually decreased. Darunavir is very soluble in polyethyleneglycol 4000. An increase in temperature accelerated the diffusion process.

After cooling the diffusion cell of the darunavir–polyethylene glycol 4000 system to room temperature, it became clear that the interdiffusion zone containing a mixture of darunavir and PEG 4000 molecules was partially crystallized. According to refractometry (Figure 4), the difference in refractive indices between darunavir and PEG 4000 was 0.1125, which corresponds to the formation of 13 interference bands in the interdiffusion zone. Plotting a concentration profile in the diffusion zone based on these data showed that the crystallization of PEG 4000 was observed at a darunavir concentration in solution of less than 15 vol.%, while above this value, the solutions were amorphous.

Thus, polyethylene glycols and related glycols were effective solvents for darunavir, offering high solubility. However, in the case of amorphous darunavir, the potential for delayed crystallization and formation of solvates, especially at elevated concentrations or during prolonged storage, should be carefully considered during formulation development and stability assessment.

##### Darunavir–PEG-40 HCO System

**Figure 14:**
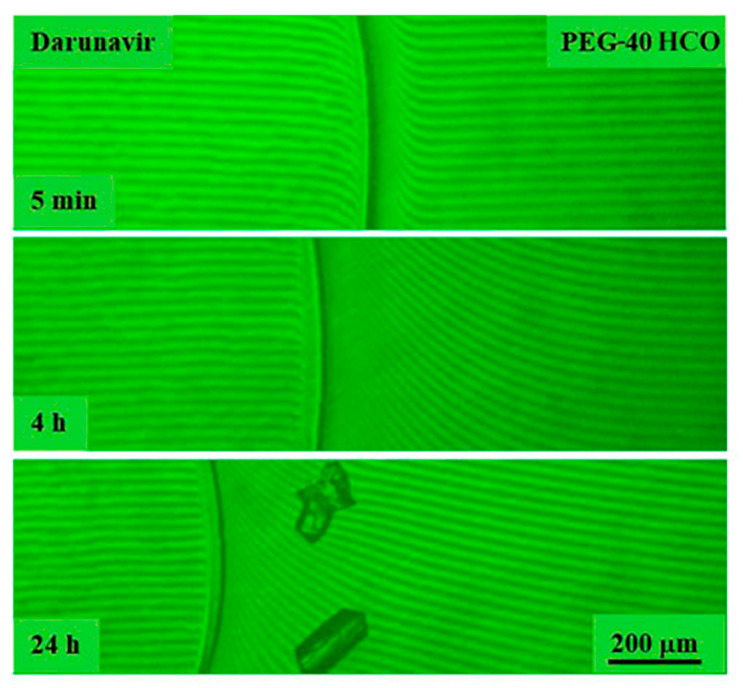
Interferograms of the darunavir–PEG-40 HCO system at 25 °C. There is no interphase boundary. Darunavir is very soluble in PEG-40 HCO. Crystal solvates were formed over time.

Dissolution studies of darunavir in ethoxlyated polyethylene glycol ester made from castor oil were also carried out in two modes: holding for 30 h at 25 °C and heating within in the temperature range of 25–130 °C. The behavior of the darunavir–PEG-40 HCO system was very similar to the darunavir–glycols system: upon contact of the components, the process of dissolution of darunavir also occurred, accompanied over time by the formation of crystalline solvates in the interdiffusion zone (Figure 14).

These findings suggest that PEG-40 HCO is an effective solvent for darunavir, making it a suitable excipient for pharmaceutical formulations, though the possibility of solvate formation should be considered during stability assessment.

##### Evaluation of Dissolution Kinetics

To compare the dissolution processes in the darunavir–alcohols, darunavir–glycols, and darunavir–PEG-40 HCO systems, the diffusion coefficients were estimated at 25 °C, as well as at 60 °C in the case of the darunavir–PEG 4000 system, in the first minutes/hours, before the formation of crystal solvates. The rate of dissolution was assessed by the movement of the front with a constant diffusant concentration. In this case, the diffusion coefficient is defined as follows:

(6) $$D=\frac{x^{2}}{6t}$$

where *x* is the diffusion coordinate and *t* is time [36]; therefore, the change in the diffusion coordinate over time is expressed as follows:

(7) $$∆x=\sqrt{6D}\cdot \sqrt{t}$$

**Figure 15:**
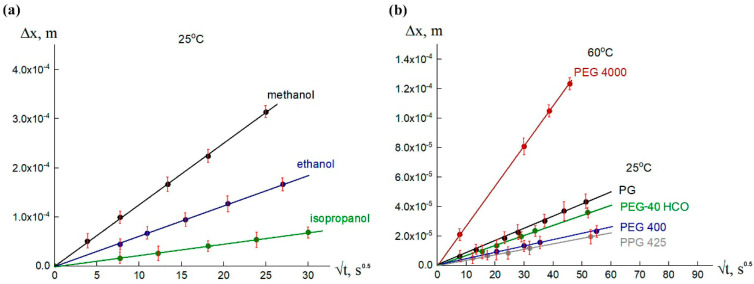
Change in diffusion coordinate over time for darunavir-alcohols (**a**) and darunavir-glycols (**b**) systems.

Figure 15 shows the time dependence of the change in diffusion coordinate for systems with alcohols and glycols, as well as with PEG-40 HCO. Based on the values of the tangent of the straight lines, the orders of the diffusion coefficients were estimated (Table 2). It can be seen that the dissolution process of darunavir occurred most quickly in methanol, 4 times slower than in ethanol, and almost 30 times slower in isopropanol. The dissolution of darunavir in glycols occurred significantly slower. The difference in diffusion coefficients with alcohols was 1–2 orders of magnitude. Increasing the temperature, as in the case of PEG 4000, brought the dissolution process closer in speed to alcohols at room temperature.

The observed changes in the diffusion coefficients when moving from one solvent to another can be explained by the corresponding changes in their viscosities. According to the Stokes–Einstein relation, the diffusion coefficient is approximately inversely proportional to the viscosity. Table 2 also provides the viscosity values of the solvents studied. It is evident that there is a direct correlation between them and the diffusion coefficients, which indirectly indicates the accuracy of the laser microinterferometry data determination.

### 3.3. Comparison of Solubility Parameters

Theoretical prediction of the solubility of substances in various solvents is of obvious practical interest. One well-known approach is the calculation of Hansen solubility parameters, which reflect the possibility of intermolecular interactions between the same or different molecules when mixed. The total strength of the various interactions is divided into dispersion (δd), polar (δp), and hydrogen components (δh), which are combined into the solubility parameter (δ), also called the three-dimensional solubility parameter. The difference in the solubility parameters of various substances (Δδ) allows us to predict their ability to be miscible with each other, i.e., dissolution. It is believed that good miscibility of components, as well as the likelihood of cocrystal formation, will be achieved if Δδ < 7 [64,65]. In the case of Δδ > 10, the substances do not mix. In this work, solubility parameters for darunavir and solvents were calculated to compare theoretical and experimental data. The criteria proposed by Hansen were used as a predictive model. Using the Y-MB module created by Dr. Yamomoto Hiroshi and based on a neural network, and the HSPiP (Hansen Solubility Parameters in Practice) program version 5.4.08, the program dataset the values of the Hansen solubility parameters and its components were determined or retrieved from the program’s internal database. [56,57]. The results are presented in Table 3.

The difference in solubility parameters in the case of the darunavir–alcohol and darunavir–glycol systems was less than 7, which suggests their good miscibility with darunavir and the possibility of forming crystal solvates. This is in good agreement with the experimental data for these systems described above. In the case of darunavir–oil systems, the difference in general solubility parameters was close to 7, but the difference in the polar component exceeded this value, which indicates poor solubility of the components and correlates with experimental data. For the systems darunavir–water and darunavir–glycerol, the values of the complete solubility parameters differed significantly, more than 10. At the same time, the difference in the values of the dispersion and polar components was very small, and the hydrogen components were very different. It is likely that it was the significant number of hydrogen bonds between solvent molecules that ensured the preference of solvent–solvent interactions compared to darunavir–solvent interactions, which is responsible for the observed low solubility of darunavir.

Of course, the theoretical calculation is not absolute, since it cannot take into account all the factors inherent in the real picture. However, the correlation of the calculated Hansen solubility parameters with the experimental data obtained by laser microinterferometry has a positive effect on the characteristics of the method. In this case, it turned out to be important to compare not only the general solubility parameters of the solute and solvent, but also their components.

## 4. Conclusions

The work demonstrates the use of laser microinterferometry to study the thermodynamic solubility of API in solvents using amorphous darunavir as an example. A wide range of commonly used solvents in pharmaceuticals were studied, including vaseline and olive oils, water, glycerol, alcohols (methanol, ethanol, isopropanol), and glycols (propylene glycol, polyethylene glycol 400, polypropylene glycol 425, polyethylene glycol 4000), as well as ethoxlyated polyethylene glycol ester made from castor oil. The phase equilibrium of most darunavir–solvent systems was studied in the temperature range of 25–130 °C, and the limits of thermodynamic solubility of the API were determined. The kinetics of the dissolution process at 25 °C were also assessed.

It was shown that darunavir is practically insoluble in olive and vaseline oils. For the darunavir–water and darunavir–glycerol systems, the presence of amorphous equilibrium with upper critical solution temperature (UCST) was revealed, phase diagrams of the systems were constructed for the first time. It was shown that darunavir can absorb up to 2.7–14 vol.% water in the temperature range of 25–95 °C, although it remained practically insoluble in water. Darunavir was slightly soluble in glycerol 1.4–7 vol.% in the temperature range of 25–130 °C, while it can absorb up to 0.4–1.2 vol.% glycerol.

In alcohols, glycols, and PEG-40 HCO, darunavir was very soluble at room temperature and above, up to 130 °C (except for systems with alcohols). Dissolution was followed by the formation of crystal solvates that melt at elevated temperatures. The rate of darunavir dissolution in methanol was the highest, in ethanol, it was 4 times slower, and in isopropanol, it was almost 30 times slower. Darunavir dissolution in glycols occurred 1–2 orders of magnitude slower than in alcohols. For the darunavir–PEG 4000 system, it was shown that the dissolution process occurred only above the melting point of the glycol. Increasing the temperature to 60 °C brought the dissolution process closer in speed to alcohols at room temperature. After cooling, it was noted that the crystallization of glycol in a solution with darunavir over 15 vol.% was disrupted.

Comparison of experimental data obtained by laser microinterferometry for the darunavir–water system with previously known data showed good correlation. Also, additional calculation of the Hansen solubility parameter values for darunavir and solvents showed good correlation of experimental data with predictions of the calculated ones.

Thus, laser microinterferometry can be successfully used in the pharmaceutical field to identify the phase state of API–solvent systems, determine the limits of thermodynamic solubility at different temperatures, detect crystal formation processes, and evaluate dissolution kinetics to understand time constraints. The ability to assess solubility, phase behavior, and dissolution kinetics across a wide temperature range within a single experiment makes laser microinterferometry a powerful tool for early-stage drug development. This comprehensive insight is highly valuable not only for the physicochemical characterization of novel compounds, but also for practical formulation tasks such as solvent selection, prediction of solid dispersion behavior (including hot-melt extrusion), determination of extrusion processing temperatures, and early risk assessment related to recrystallization or phase instability.

## Figures and Tables

**Table 1:** Refraction indices of substances at 25 °C.

Substance	*n*
Darunavir	1.594 (extrapolation)
Olive oil	1.4685
Vaseline oil	1.471
Distilled water	1.3341
Glycerol	1.4739
Methanol	1.3285
Ethanol	1.3631
Isopropanol	1.3773
PG	1.4305
PPG 425	1.4455
PEG-40 hydrogenated castor oil	1.4695
PEG 400	1.465

**Table 2:** Diffusion coefficients of systems with darunavir, as well as the viscosities of the corresponding solvents. N/A: not available.

Solvent Class	T, °C	Solvent	D, m^2^/s	η, mPa·s
Alcohols	25	Methanol	2.6 × 10^−11^	0.544 [61]
		Ethanol	6.4 × 10^−12^	1.074 [61]
		Isopropanol	8.9 × 10^−13^	2.038 [61]
Glycols	25	PG	1.2 × 10^−13^	40.4 [61]
		PEG 400	3.0 × 10^−14^	101 [62]
		PPG 425	2.2 × 10^−14^	80 [63]
	60	PEG 4000	1.2 × 10^−12^	N/A
Polyethylene glycol ester of hydrogenated castor oil	25	PEG-40 HCO	8.2 × 10^−14^	N/A

**Table 3:** Hansen solubility parameters for darunavir and various solvents.

Substance	Solubility Parameter, (MJ/m^3^)^0.5^			
	δd	δp	δh	δ
Darunavir	18.9	11.3	8.7	23.7
Olive oil	16	2.9	6.1	17.4
Vaseline oil	15.8	0.1	0.1	15.8
Water	15.5	16	42.3	47.8
Glycerol	17.4	11.3	27.2	34.2
Methanol	14.7	12.3	22.3	29.9
Ethanol	15.8	8.8	19.4	25.0
Isopropanol	15.8	6.1	16.4	21.3
PG	16.8	10.4	21.3	29.1
PPG	17.2	2.5	2.1	17.5
PEG	17.9	3.4	2.6	18.4

## Data Availability

The original contributions presented in this study are included in the article. Further inquiries can be directed to the corresponding author.

## Abbreviations

The following abbreviations are used in this manuscript:

API	Active pharmaceutical ingredient
DSC	Differential scanning calorimetry
DTT	Dissolution titration template
FDM	Facilitated dissolution method
HPLC	High-performance liquid chromatography
HSP	Hansen solubility parameters
HSPiP	Hansen solubility parameters in practice
LCST	Lower critical solution temperature
PEG 400	Polyethylene glycol 400
PEG 4000	Polyethylene glycol 4000
PEG-40 HCO	Polyethylene glycol 40 hydrogenated castor oil
PG	Propylene glycol
PPG 425	Polypropylene glycol 425
QCM	Quartz Crystal Microbalance
SPA	Single-particle analysis
SSF	Saturation shake-flask
TGA	Thermogravimetric analysis
UCST	Upper critical solution temperature
UV	Ultraviolet
μDISS	Microdissolution
